# Insect larvae, *Hermetia illucens* in poultry by-product meal for barramundi, *Lates calcarifer* modulates histomorphology, immunity and resistance to *Vibrio harveyi*

**DOI:** 10.1038/s41598-019-53018-3

**Published:** 2019-11-13

**Authors:** Md Reaz Chaklader, Muhammad A. B. Siddik, Ravi Fotedar, Janet Howieson

**Affiliations:** 1 School of Molecular and Life Sciences, Curtin University, 1 Turner Avenue, Bently, WA 6102 Australia; 2 Department of Fisheries Biology & Genetics, Faculty of Fisheries, Patuakhali Science and Technology University, Patuakhali, 8602 Bangladesh

**Keywords:** Microscopy, Antimicrobial responses, Bacterial infection, Innate immunity

## Abstract

This study investigated the effects of replacement of fishmeal (FM) with poultry by-product (PBM) protein, supplemented with black soldier fly, *Hermetia illucens* (HI) larvae on growth, histomormhology, immunity and resistance to *Vibrio harveyi* in juvenile barramundi. Two hundred and twenty five barramundi averaging 3.51 ± 0.03 g were randomly allocated into three groups and fed isonitrogenous and isocalorific diets containing different levels of PBM supplemented with HI as follows: Control (FM based diet), 45PBM + HI (45% PBM supplemented with 10% HI), and 90PBM + HI (90% PBM supplemented with 10% HI) for 6 weeks. Results showed that dietary inclusion of 45PBM + HI significantly improved the growth performance than control whereas growth inhibition occurred in the 90PBM + HI. The 45PBM + HI groups demonstrated significant increases in histometric measurements (villus and enterocyte width, and microvilli height) and acidic mucins. The impaired growth in 90PBM + HI groups was further associated with multifocal necrosis in the liver, an upregulation of the stress related genes (HSP70 and HSP90) and increase in the levels of liver enzymes. When 45PBM + HI was fed, survival against *V. harveyi* increased significantly and also an increase in serum immunity and immune-related genes in the head kidney was observed after infection.

## Introduction

For many decades, fishmeal (FM) has been used as an optimal protein source in aquafeed due to highly digestible protein, balanced amino acid composition and good palatability^1,2^. However challenges such as limited supply and increasing price of FM, burgeoning global demand for fish protein and the impact of FM production on wild marine fish stocks have motivated the aquaculture nutritionist to search and find sustainable protein rich ingredients for aquafeeds^3^. Rendered animal by-product meals consisting of discarded parts of farmed animals not being suitable for human consumption have been used for many decades in aquadiets^4^ due to the good source of amino acids, higher protein content, and energy^5–7^. PBM, a rendered by-product from the poultry processing industry can be a viable protein source to be incorporated in the diet of carnivorous fish^8^ as it has high protein content and favourable indispensable amino acid profiles^9–11^. In addition, it is a sustainable source of animal protein having a lower price than FM^12^. However, nutritional composition and digestibility of PBM vary from batch to batch and among supplier companies, which is one of the limiting factors in the utilization of PBM in aquadiets^4^. PBM as a FM replacer has been evaluated on number of marine fish species^13–19^ and success has been achieved in recent years when up to 100% PBM was able to replace FM in gilthead seabream, *Sparus aurata* L.^17^, red sea bream, *Pagrus major*^20^ and hybrid striped bass, *Morone chrysops* x *M. saxatilis*^21^. However, higher inclusion of PBM levels in the diet of fishes has also been reported to result in a number of problems including deficiencies one or more essentials amino acids (methionine and lysine)^22^, inadequate proportion of favourable fatty acids (EPA; 20:5n-3 and DHA; 22:6n-3)^23^, depressed growth performance^24^, reduced digestibility^25^ and palatability issues^22^. In particular, deficiencies of amino acids and fatty acids is one of the main shortcomings to including higher levels of PBM in the diet of carnivorous fish.

Recently, interest has turned to insects as a promising alternative protein source in aquaculture^26,27^ particularly since insects are the main prey for many omnivorous and carnivorous fish in their natural environment^28^. Insects have received attention for animal feed production when compared to conventional animal protein sources due to an ability to grow in harsh environments, often commonly infested with a wide variety of microorganisms. Such environment may induce the target insects to produce many native bioactive peptides with anti-microbial, anti-fungal and anti-viral functions^29,30^. Insects have been reported to contain biologically active antimicrobial peptides, which not only work against pathogenic bacteria, but may also boost species specific innate immune responses and promote some immunomodulatory effects^26^. Subsequently in veterinary and livestock production, these peptides have been considered as an alternative to antibiotics^31^. Nogales-Mérida, *et al*.^26^ reported that insect derivate products including protein concentrates, chitins, oils and antimicrobial peptides not only enhance the growth performance, but may also boost the fish immunity. These authors recommended to include insect meal in fish diets at very low quantities to promote fish performance and immune system function. Black soldier fly, *H. illucens* (HI) belonging to the Diptera order is one of the promising source of insect protein due to containing from 40 to 54% crude protein and 15 to 49% crude lipid (dry matter basis), well-balanced amino acids similar to FM^32,33^, and being a good source of minerals and a variety of vitamins^28^. Another important attribute of HI is the presence of antibacterial activity^30,34^ which, in low doses, may boost the immunity similarly to low doses dietary antibiotics^35^. In particular, PBM lacks certain functional components and therefore, supplementation of HI larvae with PBM could be an effective way to stimulate the growth, immunity and disease resistance against pathogens in juvenile barramundi.

Evaluation of histological changes in different organs is an effective way to assess the health status of fish. Hence, the relationship between dietary modification and the internal architecture of various tissues and cells has been a research priority in finfish production. Dietary effects of HI larvae on the histology of intestine and liver of rainbow trout, *Oncorhynchus mykiss*^1^, Jian carp, *Cyprinus carpio* var. Jian^36^ clownfish, *Amphiprion ocellaris*^37^ and Zebrafish, *Danio rerio*^38,39^ have been investigated, however, information on the histological structure of juvenile barramundi when fed HI remains largely unknown.

Barramundi, *Lates calcarifer* is a highly valued commercial species because of the fillet flavour and rapid growth^40^. It is popular for both freshwater and saltwater aquaculture and barramundi culture technology in ponds, tanks and cages is well established in Australia, Indonesia, Philippines, Malaysia, Thailand and Taiwan^41^. Intensive production of barramundi results in bacterial disease outbreaks namely Vibriosis, caused by *V. harveyi* which is the hindrance for sustainable barramundi production and causes a huge financial losses to fish farmers^42,43^. Head kidney, a large active immunocompetent organ in teleost fish contain reticular cells, macrophages, plasma cells and lymphocytes involving in antigen trapping, phagocytosis and immunologic memory^44,45^. Thus maintaining the immune function of head kidney is of importance in fish production. However, a significant effort has been given over the decades for barramundi nutrition to develop functional feed but to date, no information is available relating to the supplemental effects of HI larvae in partially or completely replacement of FM protein with PBM protein based diets. Hence, the aim of the present study was to investigate the effects of HI supplementation with PBM on growth, biometry indices, histological structure, immune response, immune-related genes and resistance to *V. harveyi* of juvenile barramundi.

## Results

### Fish performance and survival

Feeding juvenile barramundi with different levels of PBM supplemented with HI larvae significantly influenced the growth performance, biometry indices and feed utilization (Table 1). When compared to the control, growth performance in terms of FBW, SGR and WG increased significantly (*P* < 0.05) in fish fed 45PBM + HI, while growth performance was significantly lower in 90PBM + HI groups. There was no significant difference (P > 0.05) in FI between control and 45PBM + HI but decreased significantly (*P* < 0.05) in 90PBM + HI when compared with control.

**Table 1:** Fish performance, biometry indices and survival of juvenile barramundi after six weeks feeding with test diets contain various level of PBM supplemented with HI larvae.

Parameters	Test diets			ANOVA - *P*
	Control	45PBM + HI	90PBM + HI	
IBW (g)	3.48 ± 0.03^a^	3.50 ± 0.06^a^	3.53 ± 0.05^a^	0.731
FBW (g)	50.07 ± 3.54^b^	62.39 ± 2.37^a^	31.24 ± 1.92^c^	0.001
WG (g)	46.59 ± 3.57^b^	58.88 ± 2.41^a^	27.71 ± 1.90^c^	0.001
SGR (%/d)	6.33 ± 0.19^a^	6.85 ± 0.12^a^	5.18 ± 0.13^b^	0.001
FI (g/fish d^−1^)	0.78 ± 0.05^ab^	1.16 ± 0.15^a^	0.62 ± 0.63^b^	0.049
FCR	0.71 ± 0.06^a^	0.83 ± 0.07^a^	0.96 ± 0.22^a^	0.518
**Biometry indices**				
HSI (%)	1.82 ± 0.26^a^	1.52 ± 0.12^a^	1.41 ± 0.05^a^	0.043
VSI (%)	8.43 ± 0.42^a^	7.61 ± 0.44^a^	7.98 ± 0.80^a^	0.612
CF (g/cm^3^)	1.18 ± 0.05^ab^	1.48 ± 0.14^a^	1.08 ± 0.12^b^	0.224
SI (%)	0.09 ± 0.02^a^	0.10 ± 0.02^a^	0.11 ± 0.02^a^	0.667
IFI (%)	1.01 ± 0.18^ab^	0.67 ± 0.09^b^	1.12 ± 0.06^a^	0.043
RGL (%)	46.40 ± 2.85^a^	45.16 ± 2.61^a^	40.75 ± 1.77^a^	0.251

IBW = initial body weight; FBW = final body weight; WG = weight gain; SGR = specific growth rate; FI = feed intake; FCR = feed conversion ratio; HIS = hepatosomatic index; VSI = viscerasomatic index; CF = condition factor; SI = spleen index; IFI = intraperitoneal fat index and RGL = relative gut length.

Data are means ± SE. Means with different letters, within row, indicate statistical significant difference at *P* < 0.05, followed by Dunnett’s multiple comparisons test.

**Figure 1:**
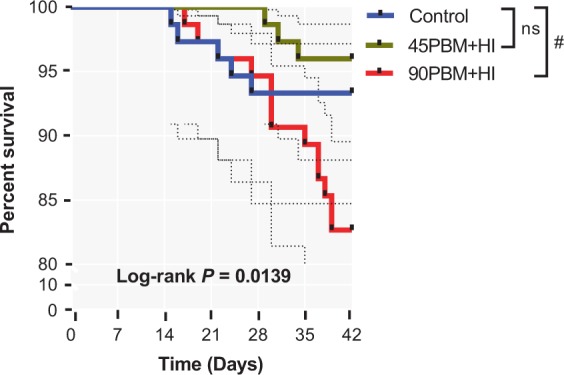
Kaplan-Meier survival rate based on Log-rank (Mantel-Cox) test of juvenile barramundi after 6 weeks feeding either control diet or different levels of PBM supplemented with HI. ^#^Indicates significant difference at *P* < 0.05.

Biometry indices including HSI, VSI, SI and RGL with the exception of CF and IFI were not influenced by different diets (Table 1). CF differed significantly (*P* < 0.05) with the lowest value observed in 90PBM + HI fed fish, whilst significantly lower value of IFI was observed in 45PBM + HI than the control and 90PBM + HI. At the end of the trial, survival rate in response to 90PBM + HI diet decreased significantly (χ^2^_90PBM+HI_ = 3.69, df = 1, *P* = 0.035) than the control, though there was no significant difference observed between control and 45PBM + HI (χ^2^_45PBM+HI_ = 0.58, df = 1, *P* = 0.447) (Fig. 1).

### Histometric measurements and histochemistry in intestine

**Figure 2:**
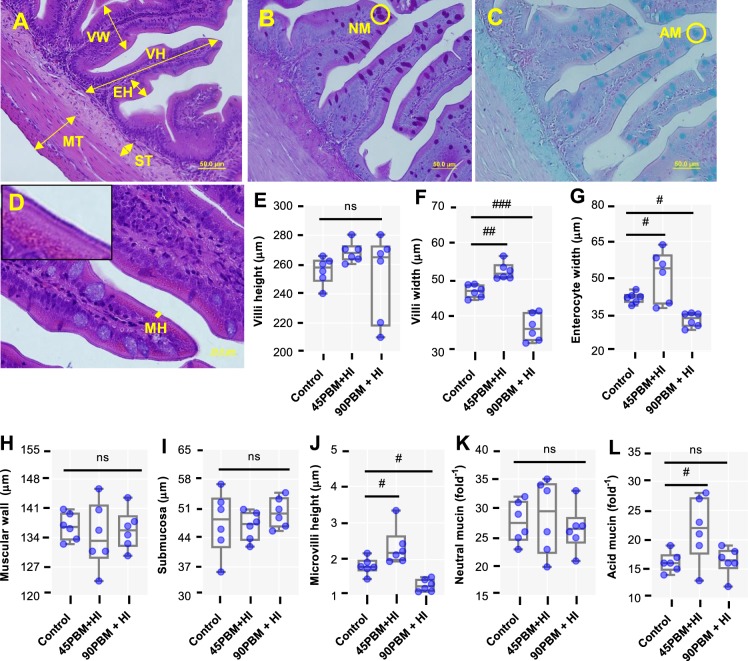
Distal intestine histometric and histochemical findings of juvenile barramundi fed control and test diets containing different levels of PBM supplemented with HI for 6 weeks. (**A**) histometric measurements (H & E stain, 40× magnification) including villi height (**E**),villi width (**F**), enterocyte width (**G**), musculosa wall (**H**) and submucosa thickness (ST); (**B**) goblet cells reacting to Periodic Acid-Schiff (PAS) stain (PAS stain, 40× magnification) represents neutral mucins (**K**); (**C**) goblet cells reacting Alcian Blue (AB) pH 2.5 represents acidic mucins (**L**); (**D**) microvilli measurements represents the height of microvilli (**J**). Data of panel (E–L) are expressed as mean ± SE (n = 6) from one representative experimental diet. ns, not significant; ^#^*P* < 0.05; ^##^*P* < 0.01 and ^###^*P* < 0.001 by Dunnett’s multiple comparisons test.

At 42 days of feeding trial, HI larvae supplementation significantly modulated the intestinal morphology and histochemistry where a significant increase in villi width (Fig. 2F) (*P* < 0.01), enterocyte width (Fig. 2G) (*P* < 0.05) and microvilli height (Fig. 2J) (*P* < 0.05) was observed in 45PBM but declined significantly in 90PBM + HI than the control. Acidic mucin per fold in 45PBM + HI increased significantly (Fig. 2L) (*P* < 0.05), though an insignificant difference was observed between control and 90PBM + HI (*P* > 0.05). However, none of the diets imposed significant effects on villi height (Fig. 2E), muscular wall thickness (Fig. 2H), submucosa thickness (Fig. 2I) and neutral mucins (Fig. 2K) (*P* > 0.05).

### Morphology of intraperitoneal adipose tissue

**Figure 3:**
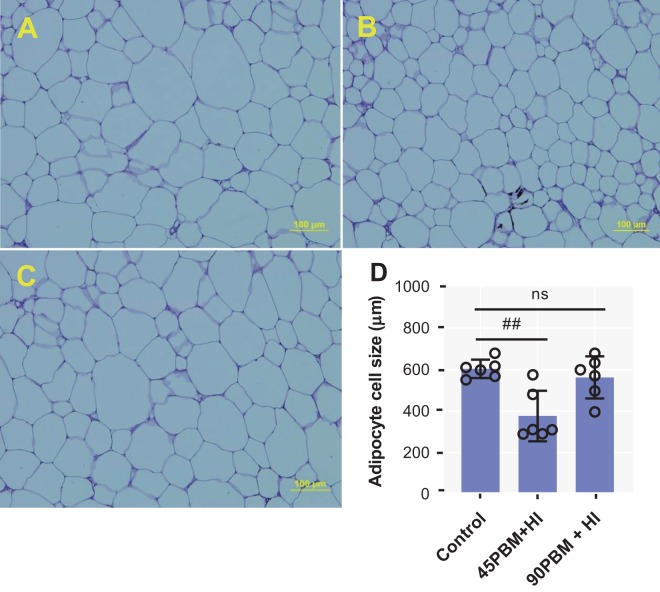
Representative adipocyte cell size structure of intraperitoneal fatty tissue of fish fed control and test diets supplemented with HI larvae after 6 weeks of feeding trial (PAS stain, 40× magnification). (**A**) intraperitoneal fatty tissue from fish fed 0% PBM and HI larvae supplementation (Control); (**B**) intraperitoneal fatty tissue from fish fed 45% PBM and 10% HI larvae supplementation (45PBM + HI); (**C**) intraperitoneal fatty tissue from fish fed 90% PBM and 10% HI larvae supplementation (90PBM + HI). (**D**) variation of adipocyte cell size of intraperitoneal fatty tissue in response to different levels of PBM supplemented with HI. Data of panel (D) are expressed as mean ± SE (n = 6) from one representative experimental diet. ns, not significant and ^##^*P* < 0.01 by Dunnett’s multiple comparisons test.

Adipocytes sizes in intraperitoneal fatty tissue in the different experimental treatments are shown in Fig. 3(A–D). The adipocytes size in fish fed 45PBM + HI decreased significantly (*P* < 0.01) than the control, whereas no significant difference was observed between control and 90PBM + HI (Fig. 3D).

### Serum biochemical indices

**Figure 4:**
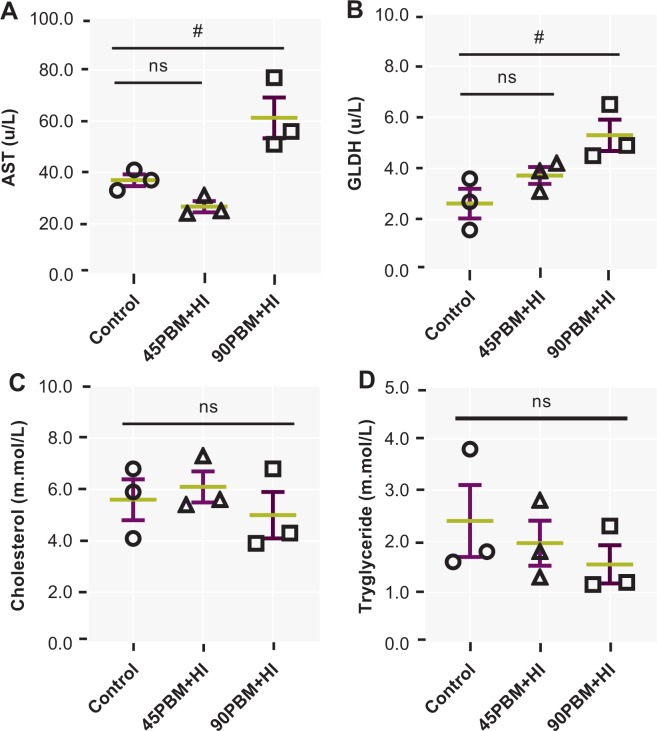
Serum AST, aspartate aminotransferase (**A**), GLDH, glutamate dehydrogenase (**B**), cholesterol (**C**) and triglyceride (**D**) of juvenile barramundi after six weeks feeding with test diets containing various level of PBM supplemented with HI. Data represent means ± SE of three values per treatment. ns, not significant and ^#^*P* < 0.05 by Dunnett’s multiple comparisons testing.

Serum biochemical parameters including AST, GLDH, cholesterol and triglyceride are shown in Fig. 4(A–D). AST and GLDH increased significantly in fish fed 90PBM + HI (*P* < 0.05) while no significant difference was observed between control and 45PBM + HI (*P* > 0.05) (Fig. 4A,B). Meanwhile, fish fed control and HI supplemented PBM diets had no significant effects on cholesterol and triglyceride (*P* > 0.05) (Fig. 4C,D).

### Histopathology and expression of HSP in liver

**Figure 5:**
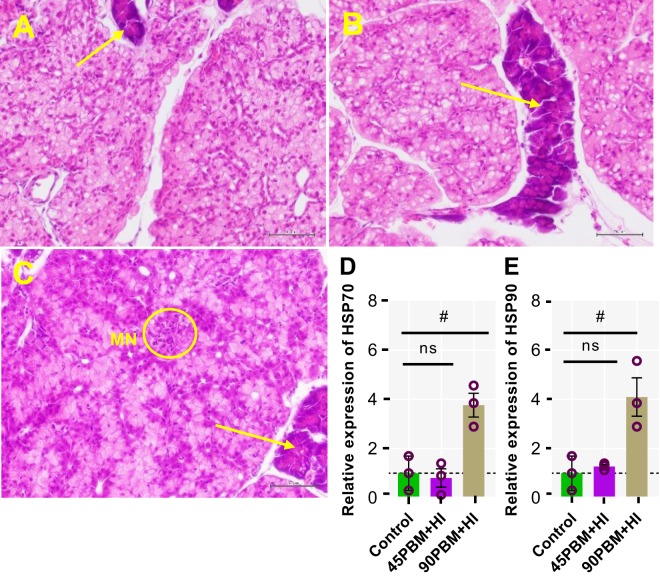
Representative histopathological features of liver sections of fish fed diets supplemented with HI larvae after 6 weeks of feeding trial (H & E stain, 40 × magnification). (**A**) liver from fish fed 0% PBM and HI larvae supplementation (Control), showing normal hepatocyte structure and exocrine pancreas with zymogen (arrow); (**B**) liver from fish fed 45% PBM and 10% HI larvae supplementation (45PBM + HI), exhibiting normal hepatocyte structure and exocrine pancreas with zymogen (arrow); (**C**) liver from fish fed 90% PBM and 10% HI larvae supplementation (90PBM + HI), revealing multifocal necrosis (yellow circle) and exocrine pancreas with zymogen (arrow). (**D**,**E**) Variation in the expression of hepatic HSP70 and HSP90 in response to different levels of PBM supplemented with HI. Data of panel (D) are expressed as mean ± SE (n = 3) from one representative experimental diet. ns, not significant and ^#^*P* < 0.05, by Dunnett’s multiple comparisons testing.

Histopathological analysis revealed that fish fed control and 45PBM + HI showed no obvious alterations with normal hepatocyte morphology and exocrine pancreas with zymogen in liver (Fig. 5A,B), however multifocal necrosis was observed in fish when fed 90PBM + HI (Fig. 5C). Both mRNA expression levels of HSP70 and HSP90 upregulated in 90PBM + HI groups (*P* < 0.05) with no significant difference between control and 45PBM + HI (*P* > 0.05) (Fig. 5D,E).

### Resistance and immunity against *V. harveyi*

**Figure 6:**
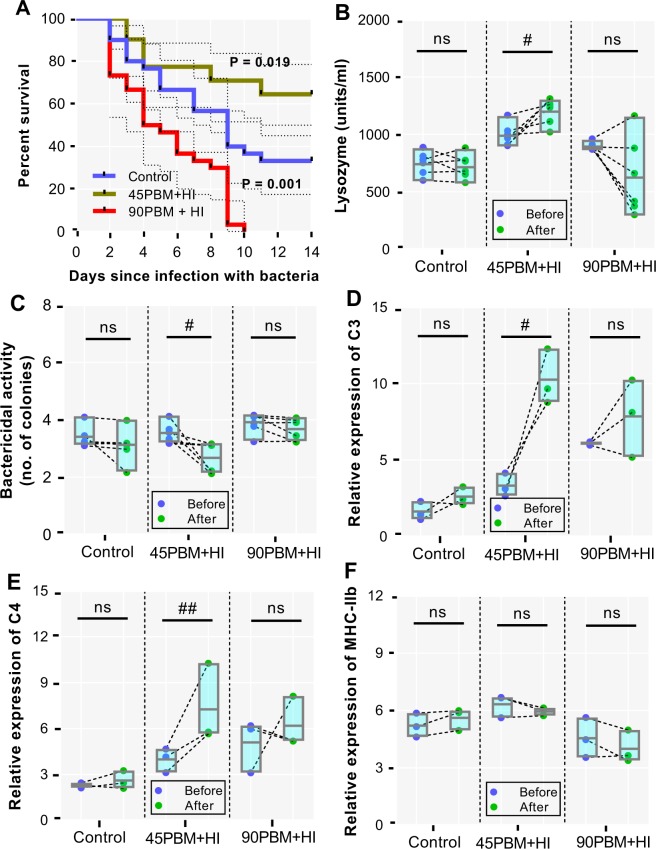
Kaplan-Meier survival (**A**) following a 14 days *V. harveyi* challenge, serum lysozyme (**B**) and bactericidal activity (**C**), relative expression of complement C3 (**D**), C4 (**E**) and MHC-IIB (**F**) in the head kidney of juvenile barramundi before and after 24 h of challenge test. Data of panel (D) are expressed as mean ± SE (n = 3) from one representative experimental diet. ns, not significant; ^#^*P* < 0.05 and ^##^*P* < 0.01 by a paired student t-test.

The survival rate of barramundi when challenged with *V. harveyi* was significantly influenced (Kaplan-Meier, log-rank (Mantel-Cox); *χ*^2^ (2) = 31.34, *P* < 0.001) by dietary supplementation of HI larvae and higher inclusion of PBM. At the end of the 14 days challenge trial, in comparison with the control, survival rate against *V. harveyi* in fish fed 45PBM + HI increased significantly (*χ*^2^_45PBM+HI_ = 5.48, df = 1, *P* = 0.019), while a significant decrease in survival rate was observed with 90PBM + HI (*χ*^2^_90PBM+HI_ = 110.71, df = 1, *P* = 0.001) (Fig. 6A). Serum lysozyme (t = 3.416, df = 10, *P* = 0.006) and bactericidal activity (t = 3.398, df = 2, *P* = 0.007) elevated in after-challenged 45PBM + HI groups when compared with before challenged 45PBM + HI groups, however there was no significant difference between before and after challenged groups fed control (t = 0.4378, df = 2, *P* = 0.6708) and 90PBM + HI diet (t = 1.940, df = 2, *P* = 0.0811) (Fig. 6B,C). Relative expression of complement C3 (t = 4.783, df = 2, *P* = 0.041) and C4 (t = 17.46, df = 2, *P* = 0.003) in response to *V. harveyi* upregulated significantly in 45PBM + HI compared to before challenge (Fig. 6D,E), while the expression levels of C3 and C4 between before and after challenged control and 90PBM + HI groups showed no significant difference. Feeding fish with control and HI larvae supplemented diet had no significant effect on MHC-IIb both in before and after challenged groups (Fig. 6F).

## Discussion

Recently, HI larvae has received much attention in aquafeed production and different inclusion levels have been successfully evaluated on variety of fish species^28^. However, no research has been carried out to investigate the supplementation of HI with PBM in the diet of any marine carnivorous species. The results of the present study revealed that supplementation of 10% HI with 45% PBM significantly improved the growth performance of juvenile barramundi when compared with control. Inclusion of HI larvae as a replacement of FM enhanced the growth performance of juvenile turbot, *Psetta maxima*^46^, yellow catfish, *Pelteobagrus fulvidraco*^47^. However, growth performance and FI decreased significantly in fish fed 90PBM + HI compared to control, which are in line with the earlier study in our lab, where inclusion of 100PBM either non- bioprocessed or bioprocessed significantly depressed the growth performance (FBG, WG and SGR) and increased FCR in juvenile barramundi, *Lates calcarifer*^48^. The possible reason for poorer growth at high PBM levels could be a reduction of palatability, deficiency in some essential amino acids and fatty acids, inconsistency in biochemical composition and low digestibility in these feeds^12,22^. Similarly, reduced palatability resulting from low feed consumption and a higher FCR have been reported in some marine carnivorous fish^14,19,49^ when fed with the diets containing higher levels of PBM (>30%). However, one of the previous studies in our laboratory was able to include 90% PBM along with 10% tuna hydrolysate supplementation in the diet of juvenile barramundi, without altering the growth performance and FCR^50^.

AST and GLDH are important liver specific enzymes in aquatic organisms, elevated rapidly in blood serum following liver cell damage and disinfection^51^. Serum AST and GLDH activity in the current study increased significantly in 90PBM + HI fed fish compare to control, implying that higher inclusion of PBM affected the functions of the liver, leading to the impairment of immune response in juvenile barramundi. However, Siddik, *et al*.^50^ reported no significant influence on serum AST and GLDH activity between control and higher replacement of PBM treated juvenile barramundi.

HI supplementation with PBM significantly influenced the IFI with significantly lower value found in 45PBM + HI, while 90PBM + HI had no significant effect on IFI in comparison with control. The lowest IFI value in 45PBM + HI might be due to dietary medium-chain fatty acids, mostly C_6_–C_12,_ which are the main fatty acids of HI meal^52^ and have been considered physiologically active compounds utilizing as an energy source and reducing the adipose tissue deposition^53^. Histological investigation of IFI can provide a valuable insight about the adipose cell size. In the current study, adipose cell size of intraperitoneal fat tissue significantly decreased in fish fed 45PBM + HI. Similar results were observed by Li, *et al*.^54^ who reported a decrease level of IFI and adipose cell size with the concurrent upregulation of mRNA level of PPARα, a lipid hydrolysis gene in juvenile Jian carp, *Cyprinus carpiovar*. Jian when fed with 75 and 100% of HI oil.

The distal intestine is an important part of the gastrointestinal tract of fish, which is more sensitive to dietary modulation and has shown highest variations in the histometric measurements including villi, microvilli and number of goblet cells (GC) in response to dietary administration of alternative protein sources^55,56^. In the present study, villi and enterocyte width and microvilli height increased significantly in fish fed 45PBM + HI, indicating the enlargement of digestion and absorption surface area. The observed shortening of villi and enterocyte width and microvilli height might be associated with poor growth performance and immune response in the 90PBM + HI. An earlier study reported that good growth performance and absorption efficiency are highly correlated with the longer villus height and width and height of microvilli^57^. Goblet cells (GCs) secret and synthesize acid and neutral mucins which are known to play an important role in lubricating, trapping and eradicating pathogens^58,59^. The presence of greater number of acidic GCs in the present study in 45PBM + HI groups signals the protection of fish by binding and preventing the adherence of pathogenic bacteria in the intestinal epithelium. A possible explanation of such positive alterations in intestine may be related to the presence of higher amount of SFA (especially lauric acid, C12) in insects, these have been demonstrated to have positive effects on gut health because of their intestinal anti-inflammatory, antiviral^38,60^ and antibacterial activity^61^.

Heat shock proteins (HSP) consisting of HSP70 and HSP90 are two important conserved cellular proteins elevate significantly in response to environmental stressors and feed factors^62,63^. Irrespective of HI supplementation, both HSP70 and HSP90 upregulated significantly when fish were fed with increasing level of PBM, revealing that higher replacement of PBM imposed stress on juvenile barramundi. Similar results were observed in histological observation in the liver of juvenile barramundi where multifocal necrosis was observed in 90PBM + HI groups. Similar to our findings, Siddik, *et al*.^48^ reported large vacuoles and irregular arrangement of liver in fish fed 100% of PBM and bioprocessed PBM.

Insects possess a wide spectrum of novel antimicrobial peptides which can exhibit activity against microbial-related disease^29^. Elhag, *et al*.^29^ identified seven new gene (cecropinZ1, sarcotoxin1, sarcotoxin (2a), sarcotoxin (2b), sarcotoxin3, stomoxynZH1,and stomoxynZH1(a)) and three types of antimicrobial peptides in HI larvae, exhibiting diverse inhibitory activity against Gram-positive bacterium, Gram-negative bacterium and fungus, suggesting a potentially important role in controlling antibiotic-resistant pathogens. Similarly, Park, *et al*.^34^ extracted low molecular weight antimicrobial factors from the HI larvae demonstrating a broad spectrum of antifungal and antibacterial activity. In the present study, fish fed 45PBM + HI diet revealed significantly higher survival rate against *V. harveyi* than other barramundi. Many earlier studies reported that dietary inclusion of insects at very low doses can enhance the disease resistance against pathogenic bacteria. For instance, Ming, *et al*.^64^ reported a higher protection rate against *Aeromonas hydrophila* in black carp, *Mylopharyngodon piceus* after 60 days of feeding 2.5% maggot, *Musca domestica* meal. In red sea bream, *Pagrus major*, dietary inclusion of 5% housefly pupae protected 100% fish from *Edwardsiella tarda* while all fish fed control diet died within 10 days following bacterial challenge^65^. Interestingly, dietary intake of HI larvae modulated the phylum of *Firmicutes* and *Proteobacteria* in the intestine of rainbow trout, *Oncorhynchus mykiss*^66^ and prebiotic bacteria in the gut of lying hens^67^. However, Li, *et al*.^68^ did not find significant difference in antibody response against infectious pancreatic necrosis virus (IPNV) between reference and HI larvae treated Atlantic salmon, *Salmo salar*. The increased resistance to *V. harveyi* might be due to the presence of chitin in HI larvae. Esteban, *et al*.^69^ and Gopalakannan and Arul^70^ reported that dietary inclusion of crustacean chitin at low levels can enhance immune response and disease resistance against pathogen in Gilthead seabream, *Sparus aurata* and common carp, *Cyprinus carpio*. However, regardless of HI supplementation, resistance to *V. harveyi* significantly declined in 90PBM + HI, revealing that higher inclusion of PBM has negative effects on immune function of barramundi. Similarly, higher replacement of FM with PBM impacted the immune function of sunshine bass, *Morone chrysops* × *Morone saxatilis*^71^ and largemouth bass, *Micropterus salmoides*^72^. Enhanced disease resistance against *V. harveyi* deserve further study to evaluate how HI larvae influence the pathogenic microbes in fish.

Several serum activities including lysozyme and bactericidal is an important indicator of innate immunity in teleost fish. Lysozyme, a low molecular weight alkaline protein is an important non-specific defense molecule in fish immune system^73,74^ and can protect the fish from the infectious disease by decomposing 1, 4 glycosidic bonds in the peptidoglycan of Gram-positive and Gram-negative cell wall^75^ and its level has been reported to be enhanced in many fish species when expose to bacterial infection^76^. In the present study, HI supplementation significantly influenced the immune response of barramundi with significantly higher values of lysozyme activity observed in 45PBM + HI treated groups after being challenged with *V. harveyi*. Similarly, serum lysozyme activity enhanced markedly in yellow catfish, *Pelteobagrus fulvidraco* when fed with 31.9% black soldier fly larvae protein^47^. Also, dietary inclusion of 10% of yellow mealworm, *Tenebrio molitor* significantly elevated lysozyme activity against Gram-negative bacteria in European sea bass, *Dicentrarchus labrax*^60^. Bactericidal activity, a nonspecific response play an important role by inhibiting and killing infectious microorganisms^50,77^. Earlier studies reported that dietary inclusion of partially defatted HI larvae can influence the beneficial bacteria in rainbow trout, *Oncorhynchus mykiss* and also HI larvae can eradicate *Escherichia coli* O157:H7 and *Salmonella enterica*^78^.

Complement system consisting of 40 difference protein molecules functioning either as enzymes or as binding proteins is an important part of innate and adaptive immune system^79^, playing a pivotal role in killing pathogens directly^80^ and promoting humoral immune responses^81^. Ohta, *et al*.^82^ identified a novel bioactive immune-activating polysaccharide composed of nine monosaccharides in the pupae of the melon fly belonging to Diptera, which induced proinflammatory cytokines and interferon β (IFNβ) in mouse against various pathogenic microorganisms and viral infections through the TLR4 signalling pathway. Complement C3 and C4 in the present study induced significantly in post-challenged 45PBM + HI groups. In line with the present study, upregulation of inflammatory genes including interleukin 1 β (*IL-1β*), *IL-8*, *IL-10* and tumor necrosis factor α (*TNF-α*) was observed in the head kidney leukocytes of salmon fed 66 and 100% of HI larvae^83^. However, the stimulation of immune response against *V. harveyi* might also be due to either secretion of aintimicrobial peptides or due to presence of chitin in insects^26,60,84^ or by other insects component^65^. Therefore, it is reasonable to hypothesize that supplementation of HI larvae with PBM can modulate the immune response of juvenile barramundi.

To conclude, PBM supplemented with 10% HI larvae effectively replaced 45% FM in feed for juvenile barramundi, supporting good growth with positive effects on histometric measurements. This results is also further supported by decrease in the size of adipose tissue cell size, enhancement of disease resistance against *V. harvei*, and elevated level of serum immune response (lysozyme and bactericidal activity) and complement systems (C3 and C4) following 24 hours of challenge test. However, 90PBM + HI significantly depressed the growth performance, reflected by the elevation of AST and GLDH activity and presence of multifocal necrosis in liver. The supplementation of HI larvae influenced the health of juvenile barramundi. However, the specific role(s) of chitin, antimicrobial peptide and/or bioactive polysaccharides of HI larvae on fish health need further investigations.

## Materials and Methods

### Ethics statements

The experiment was performed in a recirculatory aquaculture system (RAS) at Curtin Aquatic Research Laboratory (CARL) in Curtin University, Australia in accordance with the Australian Code of Practice for the care and use of animals for scientific purposes, and all protocols was reviewed and approved by the Curtin University Animal Ethics Committee (ARE2018-37). During fish handling, AQUI-S^®^ (8 mg/L) was used for anaesthesia and an overdose of AQUI-S^®^ (175 mg/L) was used as euthanasia to achieve humane endpoint when fish reached a moribund condition, following the protocol of the Curtin Research Laboratories statement of purpose (SOP) of anaesthetizing and euthanizing of fish. All experimental efforts were dedicated to minimise stress, pain and discomfort to the fish.

### Diets

Three test diets to be nearly isonitrogenous (47% crude protein) and isolipidic (13% crude lipid) containing different levels of PBM and supplemented with HI larvae meal (Table 2) were formulated from the ingredients procured from Specialty Feeds, Glen Forrest Stockfeeders, 3150 Great Eastern Highway, Glen Forrest, Western Australia 6071. The control diet contained 100% FM and the other two diets contained 45 and 90% PBM along with the 10% of HI larvae supplementation, abbreviated as 45PBM + HI and 90PBM + HI, respectively. All test diets were pelletized to 2.5 mm long and packed by Specialty Feeds, delivered to CARL and stored at −20 °C prior to the commencement of the trial. Trial ingredients and formulation are summarized in Table 2.

**Table 2:** Ingredients formulation and proximate composition of three test diets fed to juvenile barramundi for 6 weeks

*Ingredients*^a^	Test diets		
	Control	45PBM + HI	90PBM + HI
PBM^b^	0.00	31.00	63.00
Canola oil	1.00	2.40	3.00
HI larvae (full-fat)^c^	0.00	12.00	12.00
FM^d^	72.00	33.00	0.00
Corn/wheat starch	7.00	7.00	7.00
Lecithin - Soy (70%)	1.00	1.00	1.00
Vitamin C	0.05	0.05	0.05
Dicalcium Phosphate	0.05	0.05	0.05
Wheat (10 CP)	16.90	12.00	9.90
Vitamin premix	0.50	0.50	0.50
Salt (NaCl)	1.00	1.00	1.00
Cod liver oil	0.50	0.00	2.50
***Proximate composition (% dry weight)***			
Crude protein	47.88	47.86	47.97
Crude lipid	12.59	12.33	12.03

^a^Specialty Feeds, Glen Forrest Stockfeeders, 3150 Great Eastern Highway, Glen Forrest, Western Australia 6071.

^b^PBM, poultry by-product meal: crude protein (67.13%), crude lipid (13.52%) and ash (13.34%).

^c^HI, *Hermetia illucens* larvae: crude protein (40.43%) and crude lipid (17.23%).

^d^FM, fishmeal: crude protein (64.0%), crude lipid (10.76%) and ash (19.12%).

### Fish husbandry and management

**Figure 7:**
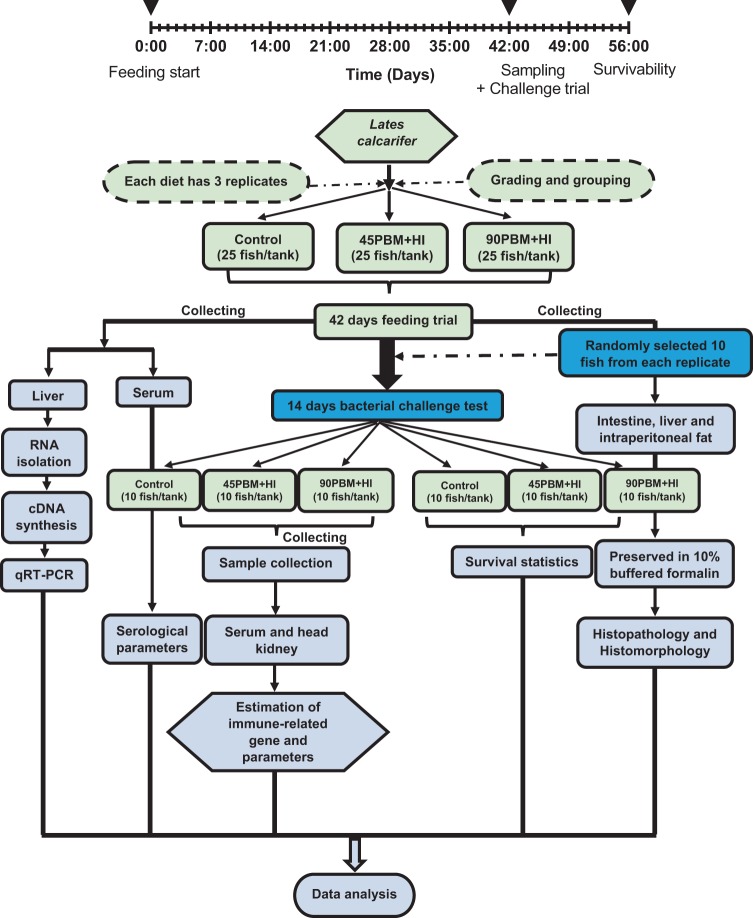
The framework represents the experimental design and analysis procedure after 42 days of feeding trial and 14 days of challenge trial.

The experimental design and analytical procedures are presented in Fig. 7. A total of 350 barramundi (mean weight = 1 g) were provided by the Australian Centre for Applied Aquaculture Research (ACAAR), Fremantle, Australia, transported in an oxygenated plastic bag and adapted to the rearing condition and facilities for two weeks at CARL in 300 L seawater tanks. The fish were fed a commercially formulated diets (470 g protein kg^−1^ and 20.0 MJ kg^−1^ gross energy) three times daily. Following acclimatization and size grading, 225 juvenile barramundi weighing 3.40 ± 0.03 g (mean ± SEM) were randomly split into 9 independent tanks (25 fish/tank). An aerator and electric heater was equipped with each tank and also an external bio-filter (Astro® 2212, China) was set up to filter the water. Water quality parameters in term of temperature (27.90–29.20 °C), dissolved oxygen (5.92–7.42 mgL^−1^), salinity (32–36 ppt), ammonia nitrogen (<0.50 mgL^−1^) and nitrite (<0.50 mgL^−1^) were checked every day and maintained within the range suitable for barramundi production^57^. Fish were reared under a 14:10 h light: dark, with the light period from 08:00 am to 10:00 pm using an automatic indoor timer (Clipsal, Australia). Each diet were hand fed in triplicate until apparent satiation twice a day (8.00 am and 6.00 pm) for 6 weeks. In order to calculate feed intake, uneaten feed was collected by siphoning from the bottom of the tank one hour after each meal, oven dried in aluminium cups for 36 h at 60 °C and then weighed. Throughout the 6 weeks of trial, mortality, if any, were checked daily to assess the fish survival rate and dead fish were weighed. After 6 weeks feeding, the fish in each tank were food deprived for 24 h, anaesthetized with AQUI-S^®^ at 8 mg/L prior to bulk weigh and then individually counted to evaluate the growth performance and survival.

### Calculation

At the beginning and end of the 42 days feeding trial, all fish were weighed to nearest to 0.1 g and fish growth performance (WG, SGR, FCR and survival) and feed intake were calculated as follows:

$$\,\begin{array}{rcl}{\rm{Weight}}\,{\rm{gain}}\,({\rm{WG}},\,{\rm{g}}) & = & [\frac{{\rm{Mean}}\,{\rm{final}}\,{\rm{weight}}-{\rm{Mean}}\,{\rm{initial}}\,{\rm{weight}}}{{\rm{Mean}}\,{\rm{initial}}\,{\rm{weight}}}]\\ {\rm{Specific}}\,{\rm{growth}}\,{\rm{rate}}\,({\rm{SGR}},\, \% /{\rm{d}}) & = & [\frac{\mathrm{ln}\,({\rm{final}}\,{\rm{body}}\,{\rm{weight}})-\,\mathrm{ln}\,({\rm{pooled}}\,{\rm{initial}}\,{\rm{weight}})}{{\rm{Days}}}]\times 100\\ {\rm{Feed}}\,{\rm{intake}}\,({\rm{FI}},\,{\rm{g}}/{\rm{fish}}\,{{\rm{d}}}^{-1}) & = & [(\frac{{\rm{Dry}}\,{\rm{diet}}\,{\rm{given}}-{\rm{Dry}}\,{\rm{remaining}}\,{\rm{diet}}\,{\rm{recovered}}}{{\rm{Days}}\,{\rm{of}}\,{\rm{experiment}}})/{\rm{No}}.\,{\rm{of}}\,{\rm{fish}}]\\ {\rm{Feed}}\,{\rm{conversion}}\,{\rm{ratio}}\,({\rm{FCR}}) & = & [\frac{{\rm{Dry}}\,{\rm{feed}}\,{\rm{fed}}}{{\rm{Wet}}\,{\rm{weigth}}\,{\rm{gain}}}]\\ {\rm{Survival}}\,({\rm{SR}},\, \% ) & = & [\frac{{\rm{Final}}\,{\rm{number}}\,{\rm{of}}\,{\rm{fish}}}{{\rm{Initial}}\,{\rm{number}}\,{\rm{of}}\,{\rm{fish}}}]\times 100\end{array}$$

Nine fish per treatment (3 fish/replicate) were collected randomly to calculate biometry indices by the following formula:

$$\begin{array}{rcl}{\rm{Condition}}\,{\rm{factor}}\,({\rm{CF}},\, \% ) & = & [\frac{{\rm{Final}}\,{\rm{body}}\,{\rm{weight}}\,({\rm{g}})}{{\rm{Body}}\,{\rm{length}}\,{{\rm{cm}}}^{3}}]\times 100\\ {\rm{Hepatosomatic}}\,{\rm{index}}\,({\rm{HSI}},\, \% ) & = & [\frac{{\rm{Liver}}\,{\rm{weight}}\,({\rm{g}})}{{\rm{Whole}}\,{\rm{body}}\,{\rm{weight}}\,({\rm{g}})}]\times 100\\ {\rm{Viscerosomatic}}\,{\rm{index}}\,({\rm{VSI}},\, \% ) & = & [\frac{{\rm{Viscera}}\,{\rm{weight}}\,({\rm{g}})}{{\rm{Whole}}\,{\rm{body}}\,{\rm{weight}}\,({\rm{g}})}]\times 100\\ {\rm{Spleen}}\,{\rm{index}}\,({\rm{SI}},\, \% ) & = & [\frac{{\rm{Weight}}\,{\rm{of}}\,{\rm{spleen}}\,({\rm{g}})}{{\rm{Whole}}\,{\rm{body}}\,{\rm{weight}}\,({\rm{g}})}]\times 100\\ {\rm{Intraperitoneal}}\,{\rm{fat}}\,{\rm{index}}\,({\rm{IFI}},\, \% ) & = & [\frac{{\rm{Intraperitoneal}}\,{\rm{fat}}\,{\rm{weight}}\,({\rm{g}})}{{\rm{Whole}}\,{\rm{body}}\,\mathrm{weight}\,({\rm{g}})}]\times 100\\ {\rm{Relative}}\,{\rm{gut}}\,{\rm{length}}\,({\rm{RGL}},\, \% ) & = & [\frac{{\rm{Length}}\,{\rm{of}}\,{\rm{intestine}}\,({\rm{cm}})}{{\rm{Length}}\,{\rm{of}}\,{\rm{fish}}\,{({\rm{cm}})}^{3}}]\times 100\end{array}$$

### Histomorphology and histopathology

At the end of 6 weeks trial, 6 fish per treatment were selected randomly and euthanized with an overdose of AQUI-S^®^ to excise liver, intraperitoneal fat and intestine for histological analysis. Fragments of all tissue samples were fixed in 10% neutral buffered formalin, dehydrated with a series of ethanol concentrations before infiltrating in xylene and embedding in paraffin wax, and finally sectioned at approximately 5 µm using a rotary microtome machine for staining with hematoxylin and eosin (H&E) following the standard histological procedures. Histological slides were digitally photographed under a light imaging microscope (BX40F4, Olympus, Tokyo, Japan).

Intestinal section were further subjected to two different stains including Periodic Acid-Schiff (PAS) and Alcian Blue (AB) pH 2.5 staining to identify neutral mucins and acidic mucins, respectively. Both type of mucins were counted from ten intact randomly selected villi, as described earlier by Elia, *et al*.^1^. Ten intact villi were randomly selected to measure the intestine histometric in terms of villi height and width, enterocyte width, muscular wall, submucosa thickness and microvilli height and diameters of adipocyte were measured using ImageJ software.

### Challenge trial with *V. harveyi*

The pathogenic bacteria, *V. harveyi* used in the challenge trial was provided by Diagnostic and Laboratory Services, Department of Primary Industries and Regional Development (DPIRD), 3 Baron-Hay Court, South Perth WA 6151. Cultures were incubated for 24 h at 24 °C in trypticase soy broth (Oxoid, Basingstoke, UK) culture medium. The culture was centrifuged for 15 minute at 5000 g and the discarded pellets were suspended in PBS (phosphate-buffered saline, pH 7.2) for challenge trail. The bacterial suspension was adjusted to 5.4 × 10^7^ cells/mL for challenge test.

The challenge trail in this study were conducted following an established CARL protocol^50^. Briefly, after 6 weeks of growth trial, 10 fish from each treatment group including the control maintaining the same replicate were selected at random and distributed to another 12 glass aquaria having 100 L capacity. Out of 12 aquaria, 9 were used to analyse the probability of survival and the remaining 3 were utilized for blood collection. Following acclimation for three days, fish were injected intraperitoneally by using a 1-mL syringe and 27-gauge needle with a lethal dose of 0.1 mL of *V. harveyi* suspension containing 5.4 × 10^7^ cells/mL and fed with same experimental diets once daily after returning the challenged fish into the respective aquaria. The signs of fish infection were monitored and recorded three times a day (7:00 am, 2:00 pm and 9:00 pm) for 14 days and infected fish were subjected to euthanasia with AQUI-S^®^ at 175 mg/L for 20 minutes according to the protocol of the CARL SOP of euthanizing of fish.

### Blood and serological parameters

At the end of the feeding and challenge trial, blood and serum samples were collected to determine biochemical and immunological indices. Six fish from each replicate (18 fish/treatment) were anaesthetized randomly using AQUI-S^®^ (8 mg l^−1^) and blood from these fish were collected from the caudal vein using 1 mL heparinized and non-heparinized syringes and pooled. Then blood in non-heparinized tubes were kept in room temperature for 24 h until coagulation followed by centrifugation (3000 rpm, 15 min) at 4 °C to obtain serum and stored at − 80 °C for the analysis of immunological indices such as lysozyme and bactericidal activity, and serum biochemical indices including aspartate aminotransferase (AST), glutamate dehydrogenase (GLDH), triglyceride and cholesterol.

Both serum lysozyme and bactericidal activity were analysed in before-challenged and after-challenged fish, following the method earlier described in Le and Fotedar^85^. Serum biochemical indices including AST, GLDH, TG, and cholesterol were assessed according to the method of Siddik, *et al*.^50^.

### RNA extraction and qRT-PCR analysis

Liver and head kidney tissues from six euthanized (AQUI-S^®^, 175 mg l^−1^) fish (two/replicate) each from control and HI supplemented groups were harvested after 42 days feeding of the trial and 24 h post infection, preserved in RNA Later (Sigma-Aldrich, Germany) and stored at − 80 °C prior to RNA extraction. For RNA extraction, the harvested frozen tissues were thawed, homogenized and ground into a fine powder. The total RNA was extracted with RNeasy Mini Kit (Qiagen, Hilden, Germany) from approximately 5 mg of various tissue samples and RNase free DNase-I (Qiagen, Hilden, Germany) was used to treat extracted RNA to remove DNA contamination. After checking the quality and quantity of RNA with gel electrophoresis and NanoDrop spectrophotometer 2000c (Thermo Fisher Scientific, USA), 1 µg of RNA for each sample (liver and head kidney) was used to synthesize complementary DNA (cDNA) Omnicript RT kit (Qiagen, Hilden, Germany) following the instruction of manufacturer’s company. qRT-PCR was carried out using PowerUp^TM^ Cyber Green Master Mix (Thermo Scientific, USA) with 7500 Real-Time PCR System (Applied Biosystems, USA) and mRNA expression level of selected genes were normalised to the *18S rRNA* and *Ef1-a*, housekeeping genes (Table 3) and calculated using 2^−ΔΔct^ method.

**Table 3:** Primers of qPCR used in the experiment.

Genes	Sequences (5ʹ-3ʹ)	Product size	Tm (°C)	
Heat shock protein kDa70, HSP70	F: AAGGCAGAGGATGATGTC R: TGCAGTCTGGTTCTTGTC	186	59	Mohd-Shaharuddin, *et al*.^86^
Heat shock protein kDa90, HSP90	F: ACCTCCCTCACAGAATACC R: CTCTTGCCATCAAACTCC	197	59	Mohd-Shaharuddin, *et al*.^86^
Complement 3, C3	F:GCAATCCTCCACAACTACAG R: ACTCTGACCTCCTGACGATAC	11	59	Mohd-Shaharuddin, *et al*.^86^
Complement 4, C4	F: TTGCTTCTTCCCTACAGTG R: GGTCCAACCCTCCTTTAC	185	59	Mohd-Shaharuddin, *et al*.^86^
MHC class IIb, MHC-IIb	F: GTTGGATACACTGAGTTTGG R: GAGGGTTTGACTGACTTAGAC	152	60	Mohd-Shaharuddin, *et al*.^86^
18S rRNA, 18S	F:TGGTTAATTCCGATAACGAACGA R: CGCCACTTGTCCCTCTAAGAA	94	59/60	Mohd-Shaharuddin, *et al*.^86^
Elongation factor-1α, ef1α	F: AAATTGGCGGTATTGGAAC R:GGGAGCAAAGGTGACGAC	83	59/60	Mohd-Shaharuddin, *et al*.^86^

### Statistical analysis

Group of fish/tank were used as experimental unit for data on growth, while individual fish were used as experimental unit for data on biometry indices, histomorphology, serum biochemical parameters, immune parameters and gene expression, as no tank-relevant effect was observed during the trial. The results of growth performance, biometry indices, histomorphology and immune parameters were expressed as mean ± standard error of mean, and subjected to normality and homogeneity of variances with Shapiro-Wilk’s and Levene’s tests. When both tests were satisfied, an ordinary one-way ANOVA with Dunnett’s multiple comparisons test was applied to test the statistical significant difference at 0.05 < *P* < 0.001 where diet was used as explanatory variable. Survival curve of barramundi at the end of the trial and after being challenged with *V. harveyi* were estimated by Kaplan-Meier method, followed by pairwise multiple comparison Log-Rank (Mantel-Cox) test. A paired student t-test was used to determine the significant difference between before and after challenge test groups.

## Data Availability

All datasets generated during the present study have been presented in the form of figures and tables but are available from the corresponding author on reasonable request.

## References

[1] Elia AC (2018). Influence of Hermetia illucens meal dietary inclusion on the histological traits, gut mucin composition and the oxidative stress biomarkers in rainbow trout (Oncorhynchus mykiss). Aquaculture.

[2] Oliva-Teles, A., Enes, P. & Peres, H. In *Feed and Feeding Practices in Aquaculture* (ed. Allen Davis, D.) 203–233 (Woodhead Publishing, 2015).

[3] FAO. The State of World Fisheries and Aquaculture 2016. Contributing to food security and nutrition for all. *The State of World Fisheries and Aquaculture* (2016).

[4] Lewis MJ (2019). A comparison of *in-vivo* and *in-vitro* methods for assessing the digestibility of poultry by-product meals using barramundi (lates calcarifer) impacts of cooking temperature and raw material freshness. Aquaculture.

[5] Bureau DP, Harris AM, Cho CY (1999). Apparent digestibility of rendered animal protein ingredients for rainbow trout (Oncorhynchus mykiss). Aquaculture.

[6] Zhou QC, Tan BP, Mai KS, Liu YJ (2004). Apparent digestibility of selected feed ingredients for juvenile cobia Rachycentron canadum. Aquaculture.

[7] Zhou Q-C, Zhao J, Li P, Wang H-L, Wang L-G (2011). Evaluation of poultry by-product meal in commercial diets for juvenile cobia (Rachycentron canadum). Aquaculture.

[8] Shapawi R, Ng W-K, Mustafa S (2007). Replacement of fish meal with poultry by-product meal in diets formulated for the humpback grouper, Cromileptes altivelis. Aquaculture.

[9] Tacon AGJ (1993). Feed ingredients for warm water fish. Fish meal and other processed feed stuffs. FAO fisheries circular.

[10] Riche M (2015). Nitrogen utilization from diets with refined and blended poultry by-products as partial fish meal replacements in diets for low-salinity cultured Florida pompano, Trachinotus carolinus. Aquaculture.

[11] Castillo-Lopez E, Espinoza-Villegas R, Viana M (2016). In vitrodigestion comparison fromfish and poultry by-product meals from simulated digestive process at different times of the Pacific Bluefintuna, *Thunnus orientalis*. Aquaculture.

[12] Cruz-Suárez LE (2007). Replacement of fish meal with poultry by-product meal in practical diets for Litopenaeus vannamei, and digestibility of the tested ingredients and diets. Aquaculture.

[13] El-Sayed A-FM (1994). Evaluation of soybean meal, spirulina meal and chicken offal meal as protein sources for silver seabream (Rhabdosargus sarba) fingerlings. Aquaculture.

[14] Fowler LG (1991). Poultry by-product meal as a dietary protein source in fall chinook salmon diets. Aquaculture.

[15] Goto T (2001). Studies on the green liver in cultured red sea bream fed low level and non-fish meal diets: relationship between hepatic taurine and biliverdin levels. Fisheries Science.

[16] Kureshy, N., Davis, D. A. & Arnold, C. R. Partial Replacement of Fish Meal with Meat-and-Bone Meal, Flash-Dried Poultry By-Product Meal, and Enzyme-Digested Poultry By-Product Meal in Practical Diets for Juvenile Red Drum. *North American Journal of Aquaculture* **62**, 266–272, 10.1577/1548-8454(2000)062<0266:PROFMW>2.0.CO2 (2000).

[17] Nengas I, Alexis MN, Davies SJ (1999). High inclusion levels of poultry meals and related byproducts in diets for gilthead seabream Sparus aurata L. Aquaculture.

[18] Wang Y, Guo J-L, Bureau DP, Cui Z-H (2006). Replacement of fish meal by rendered animal protein ingredients in feeds for cuneate drum (Nibea miichthioides). Aquaculture.

[19] Yigit M (2006). Substituting fish meal with poultry by-product meal in diets for black Sea turbot Psetta maeotica. Aquaculture Nutrition.

[20] Takagi, S., Hosokawa, H., Shimeno, S. & Ukawa, M. Utilization of poultry by-product meal in a diet for red sea bream Pagrus major. *Nippon Suisan Gakkaishi*, 428–438 (2000).

[21] Gaylord TG, Rawles SD (2005). The modification of poultry by-product meal for use in hybrid striped bass Morone chrysops x M. saxatilis diets. J. World Aquac. Soc..

[22] Fuertes JB, Celada JD, Carral JM, Sáez-Royuela M, González-Rodríguez Á (2013). Replacement of fish meal with poultry by-product meal in practical diets for juvenile crayfish (Pacifastacus leniusculus Dana, Astacidae) from the onset of exogenous feeding. Aquaculture.

[23] Norambuena F (2015). Algae in Fish Feed: Performances and Fatty Acid Metabolism in Juvenile Atlantic Salmon. PLoS One.

[24] Rossi W, Davis DA (2012). Replacement of fishmeal with poultry by-product meal in the diet of Florida pompano Trachinotus carolinus L. Aquaculture.

[25] Tibbetts S, Milley J, Lall S (2006). Apparent protein and energy digestibility of common and alternative feed ingredients by Atlantic cod, Gadus morhua (Linnaeus, 1758). Aquaculture.

[26] Nogales-Mérida, S. *et al*. Insect meals in fish nutrition. *Reviews in Aquaculture*, xocs:firstpage xmlns:xocs=, 10.1111/raq.12281 (2018).

[27] Stamer, A. Insect proteins–a new source for animal feed. The use of insect larvae to recycle food waste in high–quality protein for livestock and aquaculture feeds is held back largely owing to regulatory hurdles., Vol. 16 676–680 (EMBO Reports, 2015). 10.15252/embr.201540528 PMC4467850 25944641

[28] Henry M, Gasco L, Piccolo G, Fountoulaki E (2015). Review on the use of insects in the diet of farmed fish: Past and future. Animal Feed Science and Technology.

[29] Elhag O (2017). Screening, Expression, Purification and Functional Characterization of Novel Antimicrobial Peptide Genes from Hermetia illucens (L.). PLoS One.

[30] Park S-I, Kim J-W, Yoe SM (2015). Purification and characterization of a novel antibacterial peptide from black soldier fly (Hermetia illucens) larvae. Developmental and Comparative Immunology.

[31] Jozefiak A, Engberg RM (2017). Insect proteins as a potential source of antimicrobial peptides in livestock production. A review. J. Anim. Feed Sci..

[32] Makkar HPS, Tran G, Heuzé V, Ankers P (2014). State-of-the-art on use of insects as animal feed. Animal Feed Science and Technology.

[33] Lock ER, Arsiwalla T, Waagbø R (2016). Insect larvae meal as an alternative source of nutrients in the diet of Atlantic salmon (Salmo salar) postsmolt. Aquaculture Nutrition.

[34] Park SI, Chang BS, Yoe SM (2014). Detection of antimicrobial substances from larvae of the black soldier fly, Hermetia illucens (Diptera: Stratiomyidae). Entomological Research.

[35] Gadde U., Kim W. H., Oh S. T., Lillehoj Hyun S. (2017). Alternatives to antibiotics for maximizing growth performance and feed efficiency in poultry: a review. Animal Health Research Reviews.

[36] Li S, Ji H, Zhang B, Zhou J, Yu H (2017). Defatted black soldier fly (Hermetia illucens) larvae meal in diets for juvenile Jian carp (*Cyprinus carpio* var. Jian): Growth performance, antioxidant enzyme activities, digestive enzyme activities, intestine and hepatopancreas histological structure. Aquaculture.

[37] Vargas-AbúNdez AJ (2019). Insect meal based diets for clownfish: Biometric, histological, spectroscopic, biochemical and molecular implications. Aquaculture.

[38] Vargas A (2018). Rearing Zebrafish on Black Soldier Fly (Hermetia illucens): Biometric, Histological, Spectroscopic, Biochemical, and Molecular Implications. Zebrafish.

[39] Zarantoniello M (2019). A six-months study on Black Soldier Fly (Hermetia illucens) based diets in zebrafish. Scientific reports.

[40] Liu Y (2018). Effect of Acute Ammonia Stress on Antioxidant Enzymes and Digestive Enzymes in Barramundi Lates calcarifer Larvae. The Israeli Journal of Aquaculture - Bamidgeh.

[41] Tian X, Qin JG (2003). A single phase of food deprivation provoked compensatory growth in barramundi Lates calcarifer. Aquaculture.

[42] Ransangan, J., Lal, T. M. & Al-Harbi, A. H. Characterization and Experimental Infection of Vibrio harveyi Isolated from Diseased Asian Seabass (Lates calcarifer). *Malaysian Journal of Microbiology*, 10.21161/mjm.03512 (2012).

[43] Talpur AD, Ikhwanuddin M (2013). Azadirachta indica (neem) leaf dietary effects on the immunity response and disease resistance of Asian seabass, Lates calcarifer challenged with Vibrio harveyi. Fish and Shellfish Immunology.

[44] Press CM, Evensen Ø, Reitan LJ, Landsverk T (1996). Retention of furunculosis vaccine components in Atlantic salmon, Salmo salar L., following different routes of vaccine administration. Journal of Fish Diseases.

[45] Dannevig BH, Lauve A, Press CM, Landsverk T (1994). Receptor-mediated endocytosis and phagocytosis by rainbow trout head kidney sinusoidal cells. Fish and Shellfish Immunology.

[46] Kroeckel S (2012). When a turbot catches a fly: Evaluation of a pre-pupae meal of the Black Soldier Fly (Hermetia illucens) as fish meal substitute — Growth performance and chitin degradation in juvenile turbot (Psetta maxima). Aquaculture.

[47] Xiao X (2018). Effects of black soldier fly (Hermetia illucens) larvae meal protein as a fishmeal replacement on the growth and immune index of yellow catfish (Pelteobagrus fulvidraco). Aquaculture Research.

[48] Siddik M, Chungu P, Fotedar R, Howieson J (2019). Bioprocessed poultry by-product meals on growth, gut health and fatty acid synthesis of juvenile barramundi, Lates calcarifer (Bloch). PLoS One.

[49] Webster CD, Thompson KR, Morgan AM, Grisby EJ, Gannam AL (2000). Use of hempseed meal, poultry by-product meal, and canola meal in practical diets without fish meal for sunshine bass (Morone chrysops × M. saxatilis). Aquaculture.

[50] Siddik Muhammad A.B., Howieson Janet, Fotedar Ravi (2019). Beneficial effects of tuna hydrolysate in poultry by-product meal diets on growth, immune response, intestinal health and disease resistance to Vibrio harveyi in juvenile barramundi, Lates calcarifer. Fish & Shellfish Immunology.

[51] Kim J-H, Kang J-C (2014). The selenium accumulation and its effect on growth, and haematological parameters in red sea bream,Pagrus major, exposed to waterborne selenium. Ecotoxicology and Environmental Safety.

[52] Han J, Hamilton JA, Kirkland JL, Corkey BE, Guo W (2003). Medium-chain oil reduces fat mass and down-regulates expression of adipogenic genes in rats. Obesity research.

[53] Hashim SA, Tantibhedyangkul P (1987). Medium chain triglyceride in early life: Effects on growth of adipose tissue. Lipids.

[54] Li S (2016). Influence of black soldier fly (Hermetia illucens) larvae oil on growth performance, body composition, tissue fatty acid composition and lipid deposition in juvenile Jian carp (Cyprinus carpio var. Jian). Aquaculture.

[55] Miao S (2018). Dietary soybean meal affects intestinal homoeostasis by altering the microbiota, morphology and inflammatory cytokine gene expression in northern snakehead. Sci Rep.

[56] Gajardo K (2017). Alternative Protein Sources in the Diet Modulate Microbiota and Functionality in the Distal Intestine of Atlantic Salmon (Salmo salar). Applied and Environmental Microbiology.

[57] Siddik MAB, Howieson J, Partridge GJ, Fotedar R, Gholipourkanani H (2018). Dietary tuna hydrolysate modulates growth performance, immune response, intestinal morphology and resistance to Streptococcus iniae in juvenile barramundi, Lates calcarifer. Sci Rep.

[58] Sklan D, Prag T, Lupatsch I (2004). Structure and function of the small intestine of the tilapia Oreochromis niloticus × Oreochromis aureus (Teleostei, Cichlidae. Aquaculture Research.

[59] Padra JT (2014). Aeromonas salmonicida binds differentially to mucins isolated from skin and intestinal regions of Atlantic salmon in an N-acetylneuraminic acid-dependent manner. Infection and immunity.

[60] Henry MA, Gasco L, Chatzifotis S, Piccolo G (2018). Does dietary insect meal affect the fish immune system? The case of mealworm, Tenebrio molitor on European sea bass, Dicentrarchus labrax. Developmental and Comparative Immunology.

[61] Gasco L, Finke M, Huis VA (2018). Can diets containing insects promote animal health?. Journal of Insects as Food and Feed.

[62] Zhang X, Pang H, Wu Z, Jian J (2011). Molecular characterization of heat shock protein 70 gene transcripts during Vibrio harveyi infection of humphead snapper, Lutjanus sanguineus. Fish Physiology and Biochemistry.

[63] Lin S (2014). Genome-wide identification of hsp40 genes in channel catfish and their regulated expression after bacterial infection. PLoS ONE.

[64] Ming J (2013). The influence of maggot meal and l-carnitine on growth, immunity, antioxidant indices and disease resistance of black carp (Mylopharyngodon piceus). Journal of the Chinese Cereals and Oils Association.

[65] Ido A (2015). Dietary effects of housefly (Musca domestica) (Diptera: Muscidae) pupae on the growth performance and the resistance against bacterial pathogen in red sea bream (Pagrus major) (Perciformes: Sparidae). Applied Entomology and Zoology.

[66] Bruni L, Pastorelli R, Viti C, Gasco L, Parisi G (2018). Characterisation of the intestinal microbial communities of rainbow trout (Oncorhynchus mykiss) fed with Hermetia illucens (black soldier fly) partially defatted larva meal as partial dietary protein source. Aquaculture.

[67] Borrelli L (2017). Insect-based diet, a promising nutritional source, modulates gut microbiota composition and SCFAs production in laying hens. Sci Rep.

[68] Li Y (2019). Gut health and vaccination response in pre-smolt Atlantic salmon (Salmo salar) fed black soldier fly (Hermetia illucens) larvae meal. Fish and Shellfish Immunology.

[69] Esteban MA, Cuesta A, Ortuño J, Meseguer J (2001). Immunomodulatory effects of dietary intake of chitin on gilthead seabream (*Sparus aurata* L.) innate immune system. Fish and Shellfish Immunology.

[70] Gopalakannan A, Arul V (2006). Immunomodulatory effects of dietary intake of chitin, chitosan and levamisole on the immune system of Cyprinus carpio and control of Aeromonas hydrophila infection in ponds. Aquaculture.

[71] Rawles S (2011). Effects of replacing fish meal with poultry byaproduct meal and soybean meal and reduced protein level on the performance and immune status of pondagrown sunshine bass (*Morone chrysops M. saxatilis*). Aquaculture Nutrition.

[72] Subhadra B, Lochmann R, Rawles S, Chen R (2006). Effect of fish-meal replacement with poultry by-product meal on the growth, tissue composition and hematological parameters of largemouth bass (Micropterus salmoides) fed diets containing different lipids. Aquaculture.

[73] Wu, C. *et al*. Effects of dietary Radix Rehmanniae Preparata polysaccharides on the growth performance, immune response and disease resistance of Luciobarbus capito. *Fish and Shellfish Immunology* (2019). 10.1016/j.fsi.2019.04.027 30991149

[74] Wang C, Liu Y, Sun G, Li X, Liu Z (2019). Growth, immune response, antioxidant capability, and disease resistance of juvenile Atlantic salmon (*Salmo salar* L.) fed Bacillus velezensis V4 and Rhodotorula mucilaginosa compound. Aquaculture.

[75] Wu T (2019). What is new in lysozyme research and its application in food industry? A review. Food Chemistry.

[76] Yao C-L (2008). The lysosome and lysozyme response in Chinese shrimp Fenneropenaeus chinensis to Vibrio anguillarum and laminarin stimulation. Journal of Experimental Marine Biology and Ecology.

[77] Yano, T. The nonspecific immune system: humoral defense. In: Iwama, G. & Nakanishi, T. (Eds), The Fish Immune System. Academic Press, California, USA,. pp.105–157. (1996).

[78] Erickson MC, Islam M, Sheppard C, Liao J, Doyle MP (2004). Reduction of Escherichia coli O157:H7 and Salmonella enterica Serovar Enteritidis in Chicken Manure by Larvae of the Black Soldier Fly. Journal of Food Protection.

[79] Gasque P (2004). Complement: a unique innate immune sensor for danger signals. Molecular Immunology.

[80] Morgan BP, Marchbank KJ, Longhi MP, Harris CL, Gallimore AM (2005). Complement: central to innate immunity and bridging to adaptive responses. Immunology Letters.

[81] Beutler B (2004). Innate immunity: an overview. Molecular Immunology.

[82] Ohta T, Ido A, Kusano K, Miura C, Miura T (2014). A Novel Polysaccharide in Insects Activates the Innate Immune System in Mouse Macrophage RAW264 Cells. PLoS ONE.

[83] Stenberg O (2019). Effect of dietary replacement of fish meal with insect meal on in vitrobacterial and viral induced gene response in Atlantic salmon (Salmo salar) head kidney leukocytes. Fish and Shellfish Immunology.

[84] Lee CG, Da Silva CA, Lee J-Y, Hartl D, Elias JA (2008). Chitin regulation of immune responses: an old molecule with new roles. Current Opinion in Immunology.

[85] Le KT, Fotedar R (2014). Bioavailability of selenium from different dietary sources in yellowtail kingfish (Seriola lalandi). Aquaculture.

[86] Mohd-Shaharuddin N, Mohd-Adnan A, Kua B-C, Nathan S (2013). Expression profile of immune-related genes in Lates calcarifer infected by Cryptocaryon irritans. Fish & shellfish immunology.

